# Clinical and economic hospital burden of acute respiratory infection (BARI) due to respiratory syncytial virus in Spanish children, 2015–2018

**DOI:** 10.1186/s12879-023-08358-x

**Published:** 2023-06-08

**Authors:** F. Martinón-Torres, M. Carmo, L. Platero, G. Drago, JL. López-Belmonte, M. Bangert, J. Díez-Domingo

**Affiliations:** 1 Genetics, Vaccines and Pediatric Infectious Diseases Research Group (GENVIP), Instituto de Investigación Sanitaria de Santiago and Universidad de Santiago de Compostela (USC), Galicia, Spain; 2 Centro de Investigación Biomédica en Red de Enfermedades Respiratorias (CIBERES), Instituto de Salud Carlos III, Madrid, Spain; 3 Translational Pediatrics and Infectious Diseases, Hospital Clínico Universitario and Universidad de Santiago de Compostela (USC), Rúa da Choupana, S/N, Santiago de Compostela, 15706 Galicia, A Coruña Spain; 4 IQVIA, Barcelona, Spain; 5 Sanofi, Madrid, Spain; 6 Sanofi, Lyon, France; 7 Vaccine Research Department, FISABIO, Valencia, Spain

**Keywords:** Respiratory syncytial virus, Bronchiolitis, Burden, Epidemiology, Children, Acute lower respiratory infection

## Abstract

Respiratory syncytial virus (RSV) is a leading cause of acute lower respiratory infection (ALRI) in children, causing frequent outpatient visits and hospitalizations. Our study aimed to describe the clinical and direct economic burden of ALRI hospitalizations related to RSV in children in Spain and the characteristics of patients and their episodes. In this retrospective study, ALRI hospitalizations in children aged < 5 years for 2015–2018 were reviewed using anonymized administrative public hospital discharge data from Spain. Three case definitions were considered: (a) RSV-specific; (b) RSV-specific and unspecified acute bronchiolitis (RSV-specific and bronchiolitis); and (c) RSV-specific and unspecified ALRI (RSV-specific and ALRI). The study reported a mean of 36,743 yearly admissions potentially due to RSV, resulting in a mean annual cost of €87.1 million. RSV-specific codes accounted for 39.2% of cases, unspecified acute bronchiolitis for 20.1%, and other unspecified ALRI codes for the remaining 40.6%. The mean hospitalization rate per 1,000 children was 55.5 in the first year of life, 16.0 in the second, and 5.4 between 24 and 59 months. A considerable proportion of cases occurred in children under two years old (> 80.4%) and even during the first year of life (> 61.7%). Otherwise healthy children accounted for 92.9% of hospitalizations and 83.3% of costs during the period. Children born preterm accounted for 1.3% of hospitalizations and 5.7% of costs. The findings revealed that RSV still contributes to a high burden on the Spanish health care system. Children under one year of age and otherwise healthy term infants accounted for most of the substantial clinical and economic burden of RSV. Current evidence potentially underestimates the true epidemiology and burden of severe RSV infection; thus, further studies focusing on the outpatient setting are needed.

**Supplementary Information:**

The online version contains supplementary material available at 10.1186/s12879-023-08358-x.

## Introduction

Respiratory syncytial virus (RSV) is one of the most common seasonal infections and a major public health burden [1, 2]. It is associated with significant morbidity and mortality worldwide, particularly in children younger than two years of age, who tend to develop more serious respiratory symptoms [2–4]. In children, RSV is the leading cause of acute lower respiratory infection (ALRI) and a major driver of outpatient visits (such as to the emergency room/primary care physician) and hospitalizations [2, 5–7]. Disease manifestations can range from mild-to-severe respiratory infections (including pneumonia and bronchiolitis) to symptoms of systemic infection (such as fever and sepsis-like presentation or apnea) [3, 8]. Moreover, children who had a severe RSV infection early in life are at a greater risk of developing recurrent wheezing, hyperreactive airways, and asthma in later life [3, 9, 10].

Current treatment strategies for RSV focus on the management of high-risk children, and supportive care remains the mainstay therapy [4, 8, 11]. Infants born preterm, infants with low birth weight, and infants with congenital or acquired immunodeficiencies or with other underlying medical conditions (including Down syndrome, interstitial lung disease, neuromuscular disease, liver disease, and inborn errors of metabolism) are considered high-risk groups, as they are more likely to be affected by severe RSV infection and have higher rates of hospitalization and death [3, 4]. Despite the increased risk of morbidity and mortality in high-risk patients, the majority of children hospitalized with RSV infection have previously been healthy (i.e., had no significant medical condition) [4, 12].

Epidemiological evidence suggests that in children and infants, RSV is a major cause of death from ALRI, after pneumococcal pneumonia and *Haemophilus influenzae* type b disease [13]. The World Health Organization estimates that, globally, RSV contributes to over 60% of acute respiratory infections in children. Furthermore, RSV is responsible for more than 80% of lower respiratory infections (LTRIs) in infants younger than one year during the peak viral season [14]. A systematic review and modeling study estimated that, globally, 33 million RSV‐associated LRTIs accounted for 3.6 million hospital admissions and 101,400 deaths annually in children aged < 5 years. In children younger than six months, RSV-associated ALRI resulted in 1.4 million hospital admissions, and 45,700 deaths were estimated as attributable to RSV [7].

In Spain, a study estimated the annual incidence of RSV bronchiolitis hospitalizations to be 1,072 and 2,413 patients per 100,000, in children up to 5 and 2 years of age, respectively [15]. A study using RSV laboratory-confirmed hospitalizations in infants < 1 year old in Valencia found hospitalization incidence rates ranging from 1,097 per 100,000 infants (in season 2014/15) to 1,593 (in season 2017/18) [16]. The same study reported a seasonality of RSV between November and March, with a greater intensity in December–January, as anticipated in temperate climates, which are more likely to experience RSV epidemics during winter [5, 12, 13, 16–18]. Mortality rates of 1.47 deaths per 100,000 in children aged < 5 years and 3.40 deaths per 100,000 in children aged < 2 years have been reported in Spain, with an average of 30 deaths per year [15]. Besides potential case fatality, Díez-Gandía et al. found that RSV was associated with a mean health-related quality of life loss in Spanish children and their parents of 38%, 32%, and 9% during the first, second, and third weeks after diagnosis, respectively [19].

Health administrative data are routinely used to assess the disease burden, and the International Classification of Diseases, Tenth Revision (ICD-10), provides more RSV‐specific diagnostic codes. Still, using only RSV‐specific codes is recognized to underestimate the real burden of RSV infections, as cases are often coded according to clinical manifestations, not necessarily specifying the infectious agent behind the need for medical care [20, 21]. A combination of RSV-specific codes with general acute bronchiolitis or other general ALRI ICD‐10 codes is increasingly supported by evidence [20, 21].

In Spain, there is a lack of updated estimates on the RSV burden, and most studies include only RSV-specific and/or acute bronchiolitis codes [12, 15, 22, 23]. Hence, additional potential RSV hospitalizations might be left out, particularly in children aged 24–59 months, among whom pneumonia is observed to be a more common diagnosis [24].

The goals of this study are to describe the potential direct burden of RSV hospitalization (taking into account different case definitions) and patient and episode characteristics in patients aged < 5 years, stratified by age and risk factors.

## Methods

### Study design

The Burden of Acute Respiratory Infections (BARI) study is a real-world evidence study assessing the clinical and economic burden of acute respiratory infections (influenza and RSV) in Spain and Portugal [25–28]. We report here the results for the burden of RSV in Spain, as measured through hospitalizations and deaths.

This observational retrospective survey was conducted using anonymized administrative data on ALRI hospitalizations in children aged < 5 years in Spain in seasons 2015/2016, 2016/2017, and 2017/2018.

Anonymized administrative data on hospitalizations were used from the IASIST Projected Hospitalization Data Base (PHDB) for Spain. Minimum Basic Datasets (MBDS) collect anonymous information from inpatient episodes, including demographic, administrative, and clinical information data—including any ICD diagnosis and procedures coded for every inpatient episode of care. The PHDB collects MBDS from 189 hospitals in the National Health System (NHS). The PHDB represents approximately 55%–65% of total NHS episodes per year. The inferential methodology is used to estimate the final universe of patients hospitalized for each specific pathology [29]. This methodology provides estimated hospitalization data for the total Spanish NHS universe, which are used in this study.

### RSV case definition

The study population included children with an RSV-related event based on hospital discharge ICD-9-MC and ICD-10-ES codes, classified either as a primary or secondary diagnosis, according to three distinct case definitions: (a) RSV-specific; (b) RSV-specific and unspecified acute bronchiolitis (RSV-specific and bronchiolitis); and (c) RSV-specific and unspecified ALRI (RSV-specific and ALRI) (Table 1). These case definitions were based on findings from Cai et al., who reported a higher sensitivity for these broader definitions than RSV-specific ICD-10 codes without sacrificing specificity [20, 28].

**Table 1:**
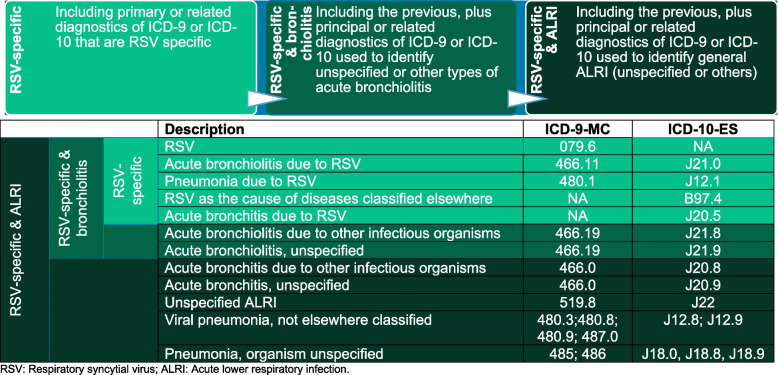
List of ICD-9/10 codes used in each case definition and frequency of observation of each code per year, in children aged < 5 years, in any primary or secondary diagnosis field

*RSV* Respiratory syncytial virus, *ALRI* Acute lower respiratory infection

### RSV case inclusion period

We extended our inclusion period from September to June to account for differences in seasonality by region. This option was made to improve the ability to detect potential RSV cases, considering the reported variability in RSV seasonality across years and countries [5, 28, 30, 31].

### Risk factors

The ICD-9-MC and ICD-10-ES diagnostic codes used to identify comorbidities/risk factors for RSV (either as primary or related diagnostics) are presented in Supplementary Table S1 (Listing S1). The following risk factors or comorbidities were considered: heart disease, respiratory failure, neuromuscular disorders, bronchopulmonary dysplasia, Down syndrome, immunodeficiency, velo-cardio-facial syndrome, congenital anomalies of the respiratory system, congenital musculoskeletal anomalies, and cystic fibrosis. Preterm birth, low birth weight, and exposure to tobacco were separately assessed, as they do not correspond to an underlying medical condition and present a higher likelihood of not being inserted by medical coders, particularly after the first hospitalization [32].

### RSV-associated in-hospital case-fatality rate

The RSV-associated in-hospital case-fatality rate was determined based on discharge status and corresponds to children who died during a hospitalization episode classified as RSV related in the numerator divided by the number of hospitalization episodes classified as RSV related in the denominator. No definitive causal link to RSV infection can be ascertained from this study.

### Other study definitions

Hospitalization incidence rates are presented as cases per 1,000 population for the overall study population, using data on age-specific annual resident population estimates downloaded from the *Instituto Nacional de Estadística* (INE, National Statistics Institute) website [33] and expressed as annual rates. Hospital incidence rates exclude the case counts from July to August for each season. The length of stay (LoS) was calculated from admission to discharge from the hospital. A respiratory severity marker was also used that comprised procedures and diagnoses such as supplementary oxygen therapy, hypoxemia, invasive and non-invasive ventilation, respiratory failure, and other abnormalities of breathing (see Supplementary Table S2 for more details).

### Cost estimation

The measure of hospital cost has been constructed using the indicator of cost per unit of hospital production (UHP). This indicator connects the operating costs incurred by the hospital to perform its activity to the production (total UHPs) carried out by the hospital. A UHP value is established for each hospital cluster in the NHS [34, 35]. The average cost per UHP is updated annually through the Hospital TOP 20 Program and considers the 3 M™ All-Patient Refined Diagnosis-Related Groups (APR DRGs) system (version 32) to calculate the degree of complexity for each hospitalization episode, considering variables related to the patient and specific episode.

### Statistical analysis

The hospitalized patients were divided into the following age groups: [0–1[ month, [1–2[ months, [2–3[ months, [3–6[ months, [6–12[ months, [12–24[ months, [24–36[ months and [36–60[ months; [0–24[ and [24–60[ months. Patient stratification with or without a relevant comorbidity (risk factor for RSV) was also carried out. Continuous data are presented as mean (standard deviation [SD]) and/or median (interquartile range [IQR]), as appropriate. Statistical analyses were carried out using IBM SPSS Statistics 19.0.

## Results

### Seasonality and birth month

**Fig. 1:**
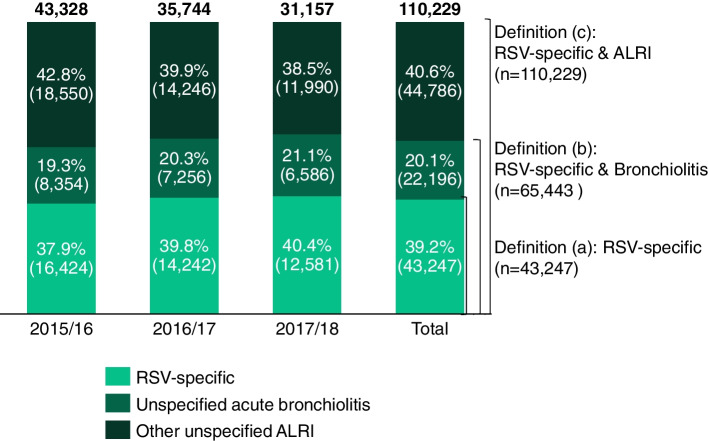
Respiratory syncytial virus-related cases in children < 5 years of age, in Spain, per groups of ICD-9/10 codes, per respiratory syncytial virus case definition and season, 2015–2018. ALRI: Acute lower respiratory infection; RSV: Respiratory syncytial virus

**Fig. 2:**
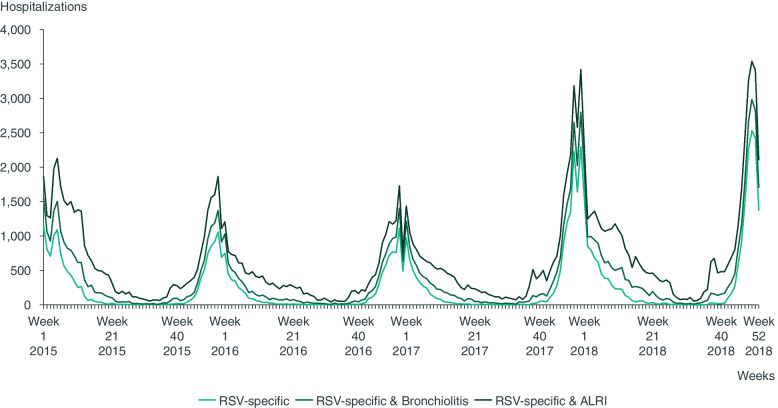
Weekly respiratory syncytial virus-related cases in children < 5 years of age between 1 January 2015 and 31st December 2018, in Spain. ALRI: Acute lower respiratory infection; RSV: Respiratory syncytial virus

A total of 110,229 RSV-specific and ALRI hospitalizations were identified during the three analyzed epidemic seasons, of which 43,328 (39.3%), 35,744 (32.4%), and 31,157 (28.3%) occurred during the 2015/2016, 2016/2017, and 2017/2018 seasons, respectively (Fig. 1). RSV-specific codes accounted for 43,247 (39.2%) cases, unspecified acute bronchiolitis codes for an additional 22,196 (20.1%) cases, and other unspecified ALRI codes for an additional 44,786 (40.6%) cases. Similar weekly trends were observed across the distinct RSV definitions, with peaks around Week 52 (Fig. 2).

**Fig. 3:**
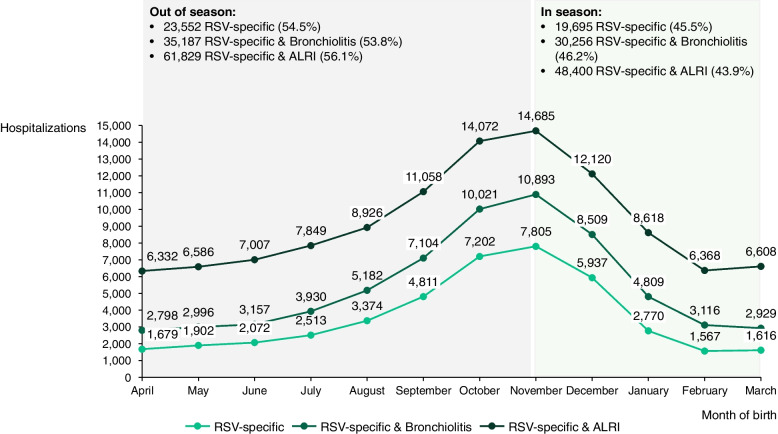
Respiratory syncytial virus-related cases in children < 5 years of age per month of birth between 1 January 2015 and 31st December 2018, per respiratory syncytial virus case definition, Spain. ALRI: Acute lower respiratory infection; RSV: Respiratory syncytial virus

Considering the month of birth, 43.9% of RSV-specific cases and ALRI and 45.5% of RSV-specific hospitalizations were observed in children who were born between November and March (Fig. 3).

### Hospitalization incidence rates

The annual incidence rate of RSV-specific and ALRI hospitalizations was highest in children with less than one year of life (55.5 per 1,000 children aged between [0–12[ months, 16.0 per 1,000 children [12–24[ months, 9.0 per 1,000 children [24–36[ months and 3.7 per 1,000 children [36–60[ months) (Listing S3). Hospitalization rates per month of age are presented in Table 2.

**Table 2:** Annual respiratory syncytial virus-associated hospitalization rate and share of hospitalizations, total and disaggregated by age groups and existence of risk factors, Spain, 2015–2018

Months of age	RSV-specific cases			RSV-specific & bronchiolitis cases			RSV-specific & ALRI cases		
	**n**	**%**	**IR**^a^	**n**	**%**	**IR**^a^	**n**	**%**	**IR**^a^
**[0–1[**	6,483	15.0	63.5	9,050	13.8	88.7	9,617	8.7	94.2
**[1–2[**	8,996	20.8	88.1	14,114	21.6	138.2	14,816	13.4	145.1
**[2–3[**	5,962	13.8	58.5	9,240	14.1	90.6	9,969	9.0	97.8
**[3–6[**	8,020	18.5	26.2	13,279	20.3	43.4	15,680	14.2	51.2
**[6–12[**	6,596	15.3	10.8	10,638	16.3	17.4	17,915	16.3	29.2
**[12–24[**	4,858	11.2	3.8	6,597	10.1	5.1	20,606	18.7	16.0
**[24–36[**	1,607	3.7	1.3	1,755	2.7	1.4	11,604	10.5	9.0
**[36–60[**	725	1.7	0.3	770	1.2	0.3	10,022	9.1	3.7
**[0–24[**	40,915	94.6	16.4	62,918	96.1	25.2	88,603	80.4	35.5
**[24–60[**	2,332	5.4	0.6	2,525	3.9	0.6	21,626	19.6	5.4
**With risk factor**	2,340	5.4	N/A	3,753	5.7	N/A	7,792	7.1	N/A
**Without risk factor**	40,907	94.6	N/A	61,690	94.3	N/A	102,437	92.9	N/A
**Total**	43,247	100.0	6.7	65,443	100.0	10.1	110,229	100.0	17.0

*ALRI* Acute lower respiratory infection, *IR* Incidence rate, *RSV* Respiratory syncytial virus, *N/A* Non-applicable

^a^The IR, or hospitalization incidence rate, is presented as registered hospitalizations per 1,000 population. The observed hospitalizations are divided by the age-specific annual resident population official estimates for each age group of the study population and multiplied by 1,000

### Demographic characteristics

A slightly higher share of hospitalizations was observed in male versus female infants regardless of the considered RSV definition (RSV-specific 55.6% versus 44.4%, RSV-specific and bronchiolitis 57.5% versus 42.5%, RSV-specific cases and ALRI 57.5% versus 42.5%). A considerable proportion of cases occurred during the first year of life (61.7% in RSV-specific cases and ALRI and 83.4% mean in RSV-specific cases) (Table 2). The median age for RSV-specific and ALRI hospitalization was 6.7 months (2.0–19.3 months) and 2.7 months (1.0–7.7 months) for RSV-specific cases.

### Risk factors

**Fig. 4:**
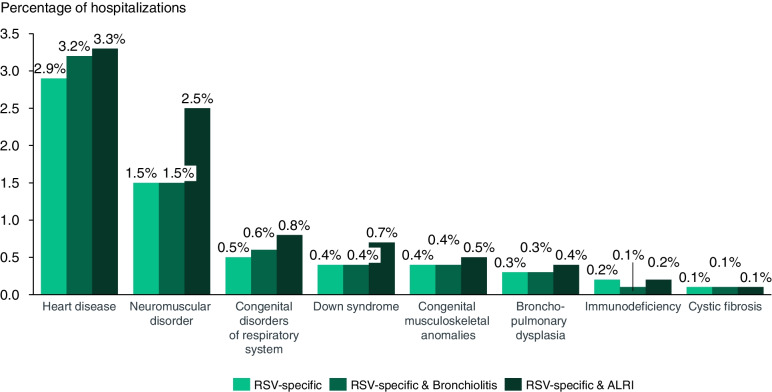
Share of relevant risk factors in respiratory syncytial virus hospitalizations per respiratory syncytial virus definition, Spain, 2015–2018. ALRI: Acute lower respiratory infection; RSV: Respiratory syncytial virus

Overall, 108,813 (98.7%) of RSV-specific and ALRI cases were observed in children born at term and 102,437 (92.9%) in children with no known predisposing risk factors for severe RSV infection. Heart disease (3.3%) was the most frequently reported risk factor, followed by neuromuscular disorders (2.5%), congenital disorders of the respiratory system (0.8%), Down syndrome (0.7%), congenital musculoskeletal anomalies (0.5%), bronchopulmonary dysplasia (0.4%), immunodeficiency (0.2%), and cystic fibrosis (0.1%) (Fig. 4). Prematurity, low birth weight, and exposure to tobacco were captured as diagnoses in 1,416 (1.3%), 1,268 (1.2%), and 82 (0.1%) cases, respectively.

### Length of stay

The mean LoS of RSV-specific and ALRI hospitalizations was 5.1 (SD 6.6) days and the median 4.0 days (2.0–6.0). The mean LoS was higher in children with a risk factor and in those born preterm or with low birth weight (Table 3).

**Table 3:** Mean length-of-stay and mean cost per patient per respiratory syncytial virus definition, stratified by age groups and existence or not of risk factors, Spain, 2015–2018

Age (months)	RSV-specific				RSV-specific & bronchiolitis				RSV-specific & ALRI			
	**LoS (days)**		**Cost/patient (€)**		**LoS (days)**		**Cost/patient (€)**		**LoS (days)**		**Cost/patient (€)**	
	**Mean**	**SD**	**Mean**	**SD**	**Mean**	**SD**	**Mean**	**SD**	**Mean**	**SD**	**Mean**	**SD**
**[0–1[**	7.9	9.5	3,588	5,249	7.5	11.4	3,370	5,052	8.8	15.6	4,137	7,279
**[1–2[**	5.7	4.5	2,276	2,134	5.4	4.9	2,142	2,100	5.4	5.6	2,166	2,176
**[2–3[**	5.4	4.3	2,158	2,657	5.2	4.7	2,052	2,496	5.3	5.2	2,092	2,604
**[3–6[**	5.3	4.6	2,112	2,134	5.0	4.5	2,029	2,063	5.3	6.1	2,171	2,857
**[6–12[**	5.0	5.1	2,260	2,344	4.7	5.2	2,163	2,154	4.7	5.4	2,261	2,375
**[12–24[**	5.0	5.6	2,348	1,934	4.6	4.7	2,226	1,802	4.2	4.7	2,278	1,786
**[24–36[**	4.8	4.8	2,454	2,607	4.7	4.8	2,391	2,532	4.1	4.6	2,332	1,982
**[36–60[**	5.3	6.1	2,959	2,960	5.2	7.8	2,912	2,909	4.1	4.9	2,395	2,189
**[0–24[**	5.7	5.9	2,437	3,024	5.4	6.3	2,239	2,817	5.3	7.4	2,417	3,330
**[24–60[**	5.0	5.3	2,612	2,790	4.9	5.9	2,551	2,718	4.1	4.7	2,360	2,095
**With risk factor**	12.3	17.0	5,696	9,458	11.2	18.2	5,049	8,360	11.4	19.9	5,614	9,170
**Without risk factor**	5.3	4.2	2,262	1,982	5.0	4.4	2,136	1,926	4.6	4.3	2,163	1,832
**Total**	5.7	5.9	2,447	3,051	5.4	5.8	2,303	2,811	5.1	6.6	2,406	3,203

*ALRI* Acute lower respiratory infection, *LoS* Length of stay, *RSV* Respiratory syncytial virus, *SD* Standard deviation

### Severity markers

A total of 52,884 (48.0%) RSV-specific and ALRI cases reported a respiratory severity marker. Invasive mechanical ventilation was used in 1,017 (0.9%) cases, non-invasive ventilation in 4,018 (3.6%), and oxygen supplementation in 26,999 (24.5%). In RSV-specific cases, a respiratory severity marker was reported in 22,467 (52.0%) hospitalizations. Invasive mechanical ventilation was used in 474 (1.1%) cases and non-invasive mechanical ventilation in 2,140 (4.9%). Oxygen supplementation was used in 11,160 (25.8.%) cases.

### In-hospital case-fatality rate

Overall, 154 deaths were reported among 110,229 RSV-specific and ALRI cases, resulting in a mean in-hospital case-fatality rate of 0.14%. Similarly, 39 deaths among 43,247 RSV-specific cases led to an in-hospital case-fatality rate of 0.09%. All deaths were observed during the first two years of life, mostly among children with at least one risk factor (72.7%). The in-hospital case-fatality rate was higher among children with at least one risk factor—RSV-specific cases and ALRI with at least one risk factor (112 [1.4%]) versus those without a risk factor (42 [0.0%])—but was overall relatively low. The median age of children who died during an RSV-specific and ALRI hospitalization was 6.0 months (2.5–18.2). Preterm birth, low weight at gestational age, and exposure to tobacco accounted for 25 (1.8% in-hospital case-fatality rate), 27 (2.1%), and 2 (2.4%) fatalities in RSV-specific and ALRI cases, respectively.

### Direct health care costs

The mean yearly total direct medical cost to the NHS for RSV-specific and ALRI hospitalizations in children aged < 5 years during the study period was €87.1 million, ranging from €75.8 million in season 2017/18 to €99.1 million in season 2015/16. Otherwise healthy children accounted for mean yearly hospitalization costs of €72.5 million (83.3%). Children born preterm accounted for 5.7% of costs.

The mean NHS hospitalization cost per patient stood at €2,406 (SD €3,203) in RSV-specific cases and ALRI. The mean cost per patient was higher in children under two years of age (€2,417, SD €3,330) than in those aged 24–59 months (€2,360, SD €2,095). The mean hospitalization cost per patient was also higher in children with a risk factor (€5,614, SD €9,170), as detailed in Table 3.

## Discussion

Using an administrative data review from 2015 to 2018, we found a high burden in Spain of hospitalizations potentially due to RSV in children aged < 5 years, especially during the first year of life. Most hospitalizations were observed in previously healthy children and in children born outside the RSV season.

To our knowledge, this is the first study reporting the burden of ALRI hospitalizations potentially related to RSV in Spain including children aged < 5 years across three seasons and distinct RSV case definitions.

The three definitions were designed to maximize the likelihood of identifying RSV cases, starting with hospitalizations classified using RSV-specific codes only, then adding unspecified acute bronchiolitis codes to the previous (RSV-specific and bronchiolitis), and, finally, adding other unspecified ALRI codes (RSV-specific cases and ALRI). Case definitions were based on findings from Cai et al. [20].

### Impact of using alternative RSV case definitions

The RSV-specific and bronchiolitis and the RSV-specific and ALRI definitions identified 1.5 and 2.5 more potential RSV cases, respectively, than RSV-specific codes alone. RSV-specific case and ALRI were estimated to represent a mean yearly cost for the NHS of €87.1 million, with RSV-specific cases accounting for 39.7% of these costs, unspecified acute bronchiolitis for 16.9%, and the other unspecified ALRI codes for the remaining 43.4%.

The RSV-specific and ALRI case definition captured a higher share of cases in children aged 24–59 months than the other two case definitions. The higher share of children aged 24–59 months in our RSV-specific and ALRI definition may be a result of the different RSV infection complications across ages. Hall et al. observed that bronchiolitis was the most frequent diagnosis in RSV-positive inpatients aged < 12 months (reported in 85% of cases), while in children aged 24–59 months, pneumonia (51%) and asthma (60%) were the most frequent diagnoses [36]. For Spain, the results across definitions are consistent with those from Martinón-Torres et al. (2022) [28]. Describing both visits to the inpatient and outpatient settings, the authors further identify differences in the pattern of required care, with 54% of RSV-specific and ALRI cases visiting emergency services and 22% being hospitalized, versus 99% and over 97% in the other two case definitions [28].

The extent to which this difference can be explained by the inclusion of non-RSV cases in the RSV-specific and ALRI definition or by a bias in RSV testing and codification toward younger children cannot be ascertained by this study [21]. Given its importance when assessing potential RSV prevention policies, investment in improved RSV diagnosis and surveillance should be made to better understand the true burden of RSV in Spain.

The RSV-specific and bronchiolitis case definition identified more potential RSV cases while maintaining a similar demographic profile to the one obtained through the RSV-specific case definition. Furthermore, this case definition has been consistently reported as having high specificity and is frequently used in studies aimed at assessing the RSV burden [20, 21, 37]. Hamilton et al. (2022) validated a set of ICD-10 algorithms in Canada to identify hospitalized patients with RSV infection [21]. They reported a 0.91 (95% CI: 0.90–0.91) positive predictive value (PPV) and 0.99 (95% CI: 0.99–0.99) specificity for RSV-specific codes. For RSV-specific and bronchiolitis, a 0.81 (95% CI: 0.80–0.82) PPV and 0.98 (95% CI: 0.98–0.98) specificity were reported. The results of this study are consistent with previous findings using similar methodologies in Spain for RSV-specific and unspecified acute bronchiolitis case definition [15].

The ability of our RSV-specific and ALRI definition to detect true RSV cases and exclude false RSV cases is expected to be more sensitive to local testing and coding practices and should be tested for Spain in future studies. Cai et al. (2020) reported 0.90 (95% CI: 0.85–0.94) specificity using this algorithm for children aged < 5 years in Germany [20]. Although Hamilton et al. did not use the same algorithm, they reported lower a PPV (0.24) and specificity (0.75) when bronchopneumonia codes (J18.0, J18.8, and J18.9) were added to RSV-specific codes [21].

### Common findings across RSV case definitions

Across all case definitions, hospitalization rates decreased as age increased. Most admissions occurred in children under two years old (80.4%–96.1%, depending on the definition) and, particularly, during the first year of life (61.7%–86.1%). The results are consistent with multiple retrospective studies from Europe that have consistently reported the highest RSV hospitalization rate in the first year of life [12, 15, 38–41]. Several studies have highlighted that 75%–90% of children hospitalized with RSV were aged ≤ 1 year and that 44%–83% were aged ≤ 6 months [39, 42–45]. Importantly, while high-risk groups may be particularly vulnerable to severe infection, we found that most hospitalizations (92.9% to 94.6%) were associated with otherwise healthy children. These findings are consistent with results from previous studies in Spain and other western countries using similar methodologies for RSV-specific and unspecified acute bronchiolitis cases [24, 36, 44, 46].

We observed a mean in-hospital case-fatality rate of 0.09% in RSV specific, 0.07% in RSV-specific and bronchiolitis, and 0.14% in RSV-specific and ALRI cases. For the equivalent case definition, we report a lower case-fatality rate than the 0.14% reported in a previous epidemiological survey in Spain in hospitalizations due to RSV bronchiolitis in children aged < 5 years of age [15]. Another regional Spanish study observed a fatality rate of 0.3% in children aged < 5 years of age [12], while yet another reported in-hospital case-fatality rates ranging between 0.07% and 0.12% in children aged < 1 year of age [47]. A study focusing on all bronchiolitis hospitalizations found similar rates (0.08%) [23]. We discovered that children who had at least one risk factor had a higher in-hospital case-fatality rate than children who did not have any risk factors, which is consistent with previous research [15, 47].

The mean LoS reported in our study is similar to that reported in previous findings, ranging from 4.0 to 5.7 days [15, 32, 48, 49]. Inpatient days gradually decrease as age increases, as reported elsewhere [50]. The LoS doubled in children with comorbidities in comparison with those without any, as previously reported for Spain [12].

The mean cost per patient was also 2.6 times higher in patients with comorbidities than in those without comorbidities, and higher in children born preterm or with low birth weight. The results are consistent with published literature [15, 51].

In terms of total cases, however, hospitalization costs were primarily driven by previously healthy term children and those aged less than two years across all RSV case definitions.

Approximately half of the hospitalized children in our study were born outside the November–March period (53.8% to 56.1%), in line with the results reported for infants hospitalized with laboratory-confirmed RSV in the Valencia region [16]. As the month of birth and age at the start of or during the RSV season are reported as statistically significant predictors of RSV hospitalization [16, 52], these data could help determine the children who may benefit the most from prevention with vaccination or monoclonal antibodies when considering immunization policies [16].

Finally, although most RSV hospitalizations were observed between December and February, in line with published dates for RSV peaks in temperate Northern Hemisphere countries [24, 40, 43, 44], the COVID-19 pandemic has been reported to have led to unexpected RSV outbreaks beginning in spring and extending into summer, further stressing the need for continued surveillance and sequencing of RSV and other respiratory viruses [53].

### Limitations

The major limitation of the present study is that it relies on data from an administrative database without linkage to RSV laboratory testing, thus not quantifying the proportion of the reported unspecified ALRI cases caused by RSV and not other infectious agents. The used case definitions were based on algorithms tested in another geography [20], and they may not necessarily hold in Spain. Although RSV is the most common pathogen identified in young children with ALRI (mainly pneumonia and bronchiolitis), other infectious agents can cause severe ALRI too [49, 54, 55]. A systematic review and meta-analysis concluded that RSV, influenza (IFV), parainfluenza (PIV), human metapneumovirus (HMPV), and rhinovirus (RV) are important causes of ALRI in young children, estimated to account for 90%, 80%, 70%, 73%, and 30% of severe ALRI cases, respectively [56].

The study is based on extrapolated administrative data, subject to coding errors or missing information. Laboratory data or drug administration data (in particular, for palivizumab) were not available. The study period included two ICD systems (ICD-9-CM and ICD-10-ES). Data on oxygenation procedures, prematurity diagnosis, weight, and tobacco exposure may have been understated. Case counts from July to August for each season were not included in the reported incidence rates to reduce the risk of including non-RSV cases. This corresponds to an exclusion of 0.2% of RSV-specific cases, 0.8% of RSV-specific and bronchiolitis cases, and 2.2% of RSV-specific and ALRI cases observed between January 2015 and December 2018.

Importantly, this study does not consider the full burden of RSV, as it includes only the burden of potential NHS RSV hospitalizations. An important burden of RSV is expected to be driven by cases treated in the outpatient setting [22, 28, 36]. In Spain, Quiles et al. reported that in Valencia, nearly 90% of bronchiolitis cases in children < 2 years were managed in outpatient settings. RSV was found to be 30 times more common in the outpatient setting in the United States than in the hospital inpatient setting [36]. Although cases treated in the private setting are not included, in 2018, only 3% of respiratory hospitalizations in children aged < 5 years old were reported as treated in private hospitals in Spain [57]. Indirect costs of lost productivity from parents and of costs due to the morbidity caused by RSV are also not considered.

## Conclusions

The findings of this study highlight that RSV represents a major burden in Spain, since it is linked with significant morbidity, particularly in young infants. Previously healthy children accounted for more than 90% of hospitalizations and 80% of hospitalization costs. The study revealed a substantial, underestimated burden of RSV, which is higher in patients aged below one year of age. The results of the BARI study showed that all infants are at risk of acquiring an RSV infection with severe complications, which are unpredictable and cause hospitalization in children without previous risk factors. There is a need to continue efforts to improve surveillance of RSV to support the introduction of prevention strategies in the future.

## Supplementary Information

**Additional file 1**: **Listing S1.** List of analyzed comorbidities and ICD9/10 codes used. **Listing S2.** List of ICD-9-MC and ICD-10-ES codes used as “severity markers”. **Listing S3.** Evolution in the incidence rate of hospitalizations by respiratory syncytial virus definition and age group.

## Data Availability

The data that support the findings of this study are available from IQVIA, but restrictions apply to the availability of these data, which were used under license for the current study and are thus not publicly available. The data are, however, available from the authors upon reasonable request and with permission from IQVIA. Those wishing to request data from this study should contact the author, Mafalda Carmo.

## References

[1] Bianchini S, Silvestri E, Argentiero A, Fainardi V, Pisi G, Esposito S (2020). Role of respiratory syncytial virus in pediatric pneumonia. Microorganisms.

[2] Eiland LS (2009). Respiratory syncytial virus: diagnosis, treatment and prevention. J Pediatr Pharmacol Ther.

[3] Stein RT, Bont LJ, Zar H, Polack FP, Park C, Claxton A, Borok G, Butylkova Y, Wegzyn C (2017). Respiratory syncytial virus hospitalization and mortality: systematic review and meta-analysis. Pediatr Pulmonol.

[4] Borchers AT, Chang C, Gershwin ME, Gershwin LJ (2013). Respiratory syncytial virus–a comprehensive review. Clin Rev Allergy Immunol.

[5] Obando-Pacheco P, Justicia-Grande AJ, Rivero-Calle I, Rodríguez-Tenreiro C, Sly P, Ramilo O, Mejías A, Baraldi E, Papadopoulos NG, Nair H (2018). Respiratory syncytial virus seasonality: a global overview. J Infect Dis.

[6] Shi T, McAllister DA, O'Brien KL, Simoes EAF, Madhi SA, Gessner BD, Polack FP, Balsells E, Acacio S, Aguayo C (2017). Global, regional, and national disease burden estimates of acute lower respiratory infections due to respiratory syncytial virus in young children in 2015: a systematic review and modelling study. Lancet (London, England).

[7] Li Y, Wang X, Blau DM, Caballero MT, Feikin DR, Gill CJ, Madhi SA, Omer SB, Simões EAF, Campbell H (2022). Global, regional, and national disease burden estimates of acute lower respiratory infections due to respiratory syncytial virus in children younger than 5 years in 2019: a systematic analysis. The Lancet.

[8] Krilov LR, Noor A, Steele RW. Respiratory Syncytial Virus Infection. In.: Medscape; 2019.

[9] Blanken MO, Rovers MM, Molenaar JM, Winkler-Seinstra PL, Meijer A, Kimpen JLL, Bont L (2013). Respiratory syncytial virus and recurrent wheeze in healthy preterm infants. N Engl J Med.

[10] Mohapatra SS, Boyapalle S (2008). Epidemiologic, experimental, and clinical links between respiratory syncytial virus infection and asthma. Clin Microbiol Rev.

[11] American Academy of Pediatrics Committee on Infectious Diseases; American Academy of Pediatrics Bronchiolitis Guidelines Committee. Updated guidance for palivizumab prophylaxis among infants and young children at increased risk of hospitalization for respiratory syncytial virus infection. Pediatrics. 2014;134(2):e620-38. 10.1542/peds.2014-1666. 10.1542/peds.2014-1666 25070304

[12] Viguria N, Martínez-Baz I, Moreno-Galarraga L, Sierrasesúmaga L, Salcedo B, Castilla J (2018). Respiratory syncytial virus hospitalization in children in northern Spain. PLoS ONE.

[13] Nair H, Nokes DJ, Gessner BD, Dherani M, Madhi SA, Singleton RJ, O'Brien KL, Roca A, Wright PF, Bruce N (2010). Global burden of acute lower respiratory infections due to respiratory syncytial virus in young children: a systematic review and meta-analysis. Lancet (London, England).

[14] Piedimonte G, Perez MK (2014). Respiratory syncytial virus infection and bronchiolitis. Pediatr Rev.

[15] Gil-Prieto R, Gonzalez-Escalada A, Marín-García P, Gallardo-Pino C, Gil-de-Miguel A (2015). Respiratory syncytial virus bronchiolitis in children up to 5 years of age in spain: epidemiology and comorbidities: an observational study. Medicine.

[16] Mira-Iglesias A, Demont C, López-Labrador FX, Mengual-Chuliá B, García-Rubio J, Carballido-Fernández M, Tortajada-Girbés M, Mollar-Maseres J, Schwarz-Chavarri G, Puig-Barberà J (2022). Role of age and birth month in infants hospitalized with RSV-confirmed disease in the Valencia Region. Spain Influenza and Other Respiratory Viruses.

[17] Servia-Dopazo M, Purriños-Hermida MJ, Pérez S, García J, Malvar-Pintos A (2020). Utilidad de la vigilancia microbiológica del virus respiratorio sincitial en Galicia (España): 2008–2017. Gac Sanit.

[18] Staadegaard L, Caini S, Wangchuk S, Thapa B, de Almeida WAF, de Carvalho FC, Fasce RA, Bustos P, Kyncl J, Novakova L (2021). Defining the seasonality of respiratory syncytial virus around the world: national and subnational surveillance data from 12 countries. Influenza Other Respir Viruses.

[19] Díez-Gandía E, Gómez-Álvarez C, López-Lacort M, Muñoz-Quiles C, Úbeda-Sansano I, Díez-Domingo J, Orrico-Sánchez A, Rigual FC, Vicent ES, Mañes C (2021). The impact of childhood RSV infection on children's and parents' quality of life: a prospective multicenter study in Spain. BMC Infect Dis.

[20] Cai W, Tolksdorf K, Hirve S, Schuler E, Zhang W, Haas W, Buda S (2020). Evaluation of using ICD-10 code data for respiratory syncytial virus surveillance. Influenza Other Respir Viruses.

[21] Hamilton MA, Calzavara A, Emerson SD, Djebli M, Sundaram ME, Chan AK, Kustra R, Baral SD, Mishra S, Kwong JC (2021). Validating international classification of disease 10th revision algorithms for identifying influenza and respiratory syncytial virus hospitalizations. PLoS ONE.

[22] Muñoz-Quiles C, López-Lacort M, Úbeda-Sansano I, Alemán-Sánchez S, Pérez-Vilar S, Puig-Barberà J, Díez-Domingo J (2016). Population-based analysis of bronchiolitis epidemiology in Valencia. Spain Pediatr Infect Dis J.

[23] Montero MH, Gil-Prieto R, Walter S, Blanquer FA, De Miguel ÁG. Burden of severe bronchiolitis in children up to 2 years of age in Spain from 2012 to 2017. Hum Vaccines Immunotherapeutics. 2022;18:1. 10.1080/21645515.2021.1883379. 10.1080/21645515.2021.1883379 PMC8920124 33653212

[24] Hall CB, Weinberg GA, Blumkin AK, Edwards KM, Staat MA, Schultz AF, Poehling KA, Szilagyi PG, Griffin MR, Williams JV (2013). Respiratory syncytial virus-associated hospitalizations among children less than 24 months of age. Pediatrics.

[25] Froes F, Carmo M, Lopes H, Bizouard G, Gomes C, Martins M, Bricout H, de Courville C, de Sousa JC, Rabaçal C (2022). Excess hospitalizations and mortality associated with seasonal influenza in Portugal, 2008–2018. BMC Infect Dis.

[26] Gil-de-Miguel Á, Martinón-Torres F, Díez-Domingo J, de Lejarazu Leonardo RO, Pumarola T, Carmo M, Drago G, López-Belmonte JL, Bricout H, de Courville C (2022). Clinical and economic burden of physician-diagnosed influenza in adults during the 2017/2018 epidemic season in Spain. BMC Public Health.

[27] Bandeira T, Carmo M, Lopes H, Gomes C, Martins M, Guzman C, Bangert M, Rodrigues F, Januário G, Tomé T (2023). Burden and severity of children's hospitalizations by respiratory syncytial virus in Portugal, 2015–2018. Influenza Other Respir Viruses.

[28] Martinón-Torres F, Carmo M, Platero L, Drago G, López-Belmonte J, Bangert M, Díez-Domingo J, Garcés-Sánchez M (2022). Clinical and economic burden of respiratory syncytial virus in Spanish children: the BARI study. BMC Infect Dis.

[29] Lindner L, García-Sánchez R, Alvarez C, Betegón L, Badia X (2013). Hospitalizaciones por hipoglucemia grave en pacientes con diabetes mellitus en España. Rev Clin Esp.

[30] Bloom-Feshbach K, Alonso WJ, Charu V, Tamerius J, Simonsen L, Miller MA, Viboud C (2013). Latitudinal variations in seasonal activity of influenza and respiratory syncytial virus (RSV): a global comparative review. PLoS ONE.

[31] Janet S, Broad J, Snape MD (2018). Respiratory syncytial virus seasonality and its implications on prevention strategies. Hum Vaccin Immunother.

[32] Mendes-da-Silva A, GonçalvesPinho M, Freitas A, Azevedo I (2018). Trends in hospitalization for acute bronchiolitis in Portugal: 2000–2015. Pulmonology.

[33] INE: Población residente por grupo de edad, a 1 enero de cada año, 2008–2019. In.: Instituto Nacional de Estadística; 2020. https://www.ine.es/jaxiT3/Tabla.htm?t=9689&L=0.

[34] García-Eroles L, Illa C, Arias A, Casas M (2001). Los Top 20 2000: objetivos, ventajas y limitaciones del método. Rev Calid Asist.

[35] Sánchez-Martínez F, Abellán-Perpiñán JM, Martínez-Pérez JE, Puig-Junoy J (2006). Cost accounting and public reimbursement schemes in Spanish hospitals. Health Care Manag Sci.

[36] Hall CB, Weinberg GA, Iwane MK, Blumkin AK, Edwards KM, Staat MA, Auinger P, Griffin MR, Poehling KA, Erdman D (2009). The burden of respiratory syncytial virus infection in young children. N Engl J Med.

[37] Pisesky A, Benchimol EI, Wong CA, Hui C, Crowe M, Belair M-A, Pojsupap S, Karnauchow T, O'Hearn K, Yasseen AS (2016). Incidence of hospitalization for respiratory syncytial virus infection amongst children in Ontario, Canada: a population-based study using validated health administrative data. PLoS ONE.

[38] Ajayi-Obe EK, Coen PG, Handa R, Hawrami K, Aitken C, McIntosh EDG, Booy R (2008). Influenza A and respiratory syncytial virus hospital burden in young children in East London. Epidemiol Infect.

[39] Resch B, Gusenleitner W, Mandl C, Müller W (2000). Epidemiology of respiratory syncytial virus infection in Southern Austria. Pediatr Infect Dis J.

[40] Vicente D, Montes M, Cilla G, Perez-Yarza EG, Perez-Trallero E (2003). Hospitalization for respiratory syncytial virus in the paediatric population in Spain. Epidemiol Infect.

[41] Heppe-Montero M, Walter S, Hernández-Barrera V, Gil-Prieto R, Gil-de-Miguel Á (2022). Burden of respiratory syncytial virus-associated lower respiratory infections in children in Spain from 2012 to 2018. BMC Infect Dis.

[42] Deshpande SA, Northern V (2003). The clinical and health economic burden of respiratory syncytial virus disease among children under 2 years of age in a defined geographical area. Arch Dis Child.

[43] Fjaerli HO, Farstad T, Bratlid D (2004). Hospitalisations for respiratory syncytial virus bronchiolitis in Akershus, Norway, 1993–2000: a population-based retrospective study. BMC Pediatr.

[44] García CG, Bhore R, Soriano-Fallas A, Trost M, Chason R, Ramilo O, Mejias A (2010). Risk factors in children hospitalized with RSV bronchiolitis versus non-RSV bronchiolitis. Pediatrics.

[45] Hervás D, Reina J, Yañez A, del Valle JM, Figuerola J, Hervás JA (2012). Epidemiology of hospitalization for acute bronchiolitis in children: differences between RSV and non-RSV bronchiolitis. Eur J Clin Microbiol Infect Dis.

[46] Boyce TG, Mellen BG, Mitchel EF, Wright PF, Griffin MR (2000). Rates of hospitalization for respiratory syncytial virus infection among children in medicaid. J Pediatr.

[47] Sanchez-Luna M, Elola FJ, Fernandez-Perez C, Bernal JL, Lopez-Pineda A (2016). Trends in respiratory syncytial virus bronchiolitis hospitalizations in children less than 1 year: 2004–2012. Curr Med Res Opin.

[48] Tsolia MN, Kafetzis D, Danelatou K, Astra H, Kallergi K, Spyridis P, Karpathios TE (2003). Epidemiology of respiratory syncytial virus bronchiolitis in hospitalized infants in Greece. Eur J Epidemiol.

[49] Bont L, Checchia PA, Fauroux B, Figueras-Aloy J, Manzoni P, Paes B, Simões EA, Carbonell-Estrany X (2016). Defining the epidemiology and burden of severe respiratory syncytial virus infection among infants and children in Western Countries. Infect Dis Ther.

[50] Jepsen MT, Trebbien R, Emborg HD, Krause TG, Schønning K, Voldstedlund M, Nielsen J, Fischer TK (2018). Incidence and seasonality of respiratory syncytial virus hospitalisations in young children in Denmark, 2010 to 2015. Eurosurveillance.

[51] Shefali-Patel D, Paris MA, Watson F, Peacock JL, Campbell M, Greenough A (2012). RSV hospitalisation and healthcare utilisation in moderately prematurely born infants. Eur J Pediatr.

[52] Mauskopf J, Margulis AV, Samuel M, Lohr KN (2016). Respiratory syncytial virus hospitalizations in healthy preterm infants: systematic review. Pediatr Infect Dis J.

[53] Eden JS, Sikazwe C, Xie R, Deng YM, Sullivan SG, Michie A, Levy A, Cutmore E, Blyth CC, Britton PN, Crawford N, Dong X, Dwyer DE, Edwards KM, Horsburgh BA, Foley D, Kennedy K, Minney-Smith C, Speers D, Tulloch RL, Holmes EC, Dhanasekaran V, Smith DW, Kok J, Barr IG; Australian RSV study group. Off-season RSV epidemics in Australia after easing of COVID-19 restrictions. Nat Commun. 2022;13(1):2884. 10.1038/s41467-022-30485-3. 10.1038/s41467-022-30485-3 PMC9130497 35610217

[54] Simoes EA (1999). Respiratory syncytial virus infection. The lancet.

[55] Simoes EA, Cherian T, Chow J, Shahid-Salles SA, Laxminarayan R, John TJ: Acute respiratory infections in children. Disease Control Priorities in Developing Countries 2nd edition 2006.

[56] Shi T, McLean K, Campbell H, Nair H (2015). Aetiological role of common respiratory viruses in acute lower respiratory infections in children under five years: a systematic review and meta–analysis. J Global Health.

[57] Sanitaria. MdSSGdI: Registro de Actividad de Atención Especializada – RAE-CMBD. In.; 2018.

