# Supplementary material for: Clinical and economic hospital burden of acute respiratory infection (BARI) due to respiratory syncytial virus in Spanish children, 2015–2018
Source: BMC Infect Dis. 2023 Jun 8;23:385. doi: 10.1186/s12879-023-08358-x (PMC10249572; doi:10.1186/s12879-023-08358-x)
Supplement: Supplementary file 1 — Additional file 1: Listing S1. List of analyzed comorbidities and ICD9/10 codes used. Listing S2. List of ICD-9-MC and ICD-10-ES codes used as “severity markers”. Listing S3. Evolution in the incidence rate of hospitalizations by respiratory syncytial virus definition and age group. [file 12879_2023_8358_MOESM1_ESM.docx]

## **Supplementary Materials**

Listing S1 List of analyzed comorbidities and ICD9/10 codes used

|  | Description | ICD-9-MC | ICD-10-ES |
| --- | --- | --- | --- |
| Preterm | Extreme immaturity of newborn, unspecified weeks of gestation | 765.20 | P07.20 |
|  | Extreme immaturity of newborn, gestational age less than 23 completed weeks | 765.21 | P07.21 |
|  | Extreme immaturity of newborn, gestational age 23 completed weeks | 765.21 | P07.22 |
|  | Extreme immaturity of newborn, gestational age 24 completed weeks | 765.22 | P07.23 |
|  | Extreme immaturity of newborn, gestational age 25 completed weeks | 765.23 | P07.24 |
|  | Extreme immaturity of newborn, gestational age 26 completed weeks | 765.23 | P07.25 |
|  | Extreme immaturity of newborn, gestational age 27 completed weeks | 765.24 | P07.26 |
|  | Preterm [premature] newborn [other] | - | P07.3 |
|  | Preterm newborn, unspecified weeks of gestation | 765.09; 765.19; 765.20 | P07.30 |
|  | Preterm newborn, gestational age 28 completed weeks | 765.24 | P07.31 |
|  | Preterm newborn, gestational age 29 completed weeks | 765.25 | P07.32 |
|  | Preterm newborn, gestational age 30 completed weeks | 765.25 | P07.33 |
|  | Preterm newborn, gestational age 31 completed weeks | 765.26 | P07.34 |
|  | Preterm newborn, gestational age 32 completed weeks | 765.26 | P07.35 |
|  | Preterm newborn, gestational age 33 completed weeks | 765.27 | P07.36 |
|  | Preterm newborn, gestational age 34 completed weeks | 765.27 | P07.37 |
|  | Preterm newborn, gestational age 35 completed weeks | 765.28 | P07.38 |
|  | Preterm newborn, gestational age 36 completed weeks | 765.28 | P07.39 |
| **Low weight or size at gestational age** | Newborn light for gestational age, unspecified weight | 764.00 | P05.00 |
|  | Newborn light for gestational age, less than 500 grams | 764.01 | P05.01 |
|  | Newborn light for gestational age, 500 - 749 grams | 764.02 | P05.02 |
|  | Newborn light for gestational age, 750-999 grams | 764.03 | P05.03 |
|  | Newborn light for gestational age, 1000-1249 grams | 764.04 | P05.04 |
|  | Newborn light for gestational age, 1250-1499 grams | 764.05 | P05.05 |
|  | Newborn light for gestational age, 1500-1749 grams | 764.06 | P05.06 |
|  | Newborn light for gestational age, 1750-1999 grams | 764.07 | P05.07 |
|  | Newborn light for gestational age, 2000-2499 grams | 764.08 | P05.08 |
|  | Newborn light for gestational age, 2500 grams and over | 764.09 | P05.09 |
|  | Newborn small for gestational age, unspecified weight | 764.10 | P05.10 |
|  | Newborn small for gestational age, less than 500 grams | 764.11 | P05.11 |
|  | Newborn small for gestational age, 500-749 grams | 764.12 | P05.12 |
|  | Newborn small for gestational age, 750-999 grams | 764.13 | P05.13 |
|  | Newborn small for gestational age, 1000-1249 grams | 764.14 | P05.14 |
|  | Newborn small for gestational age, 1250-1499 grams | 764.15 | P05.15 |
|  | Newborn small for gestational age, 1500-1749 grams | 764.16 | P05.16 |
|  | Newborn small for gestational age, 1750-1999 grams | 764.17 | P05.17 |
|  | Newborn small for gestational age, 2000-2499 grams | 764.18 | P05.18 |
|  | Newborn small for gestational age, other | 764.19 | P05.19 |
|  | Newborn affected by fetal (intrauterine) malnutrition not light or small for gestational age | 764.20 | P05.2 |
|  | Newborn affected by slow intrauterine growth, unspecified | 764.90 | P05.9 |
|  | Extremely low birth weight newborn, unspecified weight | 765.0 765.10 | P07.00 |
|  | Extremely low birth weight newborn, less than 500 grams | 765.01765.11 | P07.01 |
|  | Extremely low birth weight newborn, 500-749 grams | 765.02  765.12 | P07.02 |
|  | Extremely low birth weight newborn, 750-999 grams | 765.03  765.13 | P07.03 |
|  | Other low birth weight newborn, unspecified weight | 765.00  765.10 | P07.10 |
|  | Other low birth weight newborn, 1000-1249 grams | 765.04 765.14 | P07.14 |
|  | Other low birth weight newborn, 1250-1499 grams | 765.05  765.15 | P07.15 |
|  | Other low birth weight newborn, 1500-1749 grams | 765.06  765.16 | P07.16 |
|  | Other low birth weight newborn, 1750-1999 grams | 765.07  765.17 | P07.17 |
|  | Other low birth weight newborn, 2000-2499 grams | 765.08  765.18 | P07.18 |
| **Exposure to tobacco** | Exposure to (parental) (environmental) tobacco smoke in the perinatal period | 760.8 | P96.81 |
|  | Contact with and (suspected) exposure to environmental tobacco smoke (acute) (chronic) | V15.89; E869.4 | Z77.22 |
| Heart disease | Other chronic pulmonary heart diseases | 416.8 | I27.89; I27.2 |
|  | Other primary cardiomyopathies | 425.4 | I42.5; I42.8 |
|  | Congenital malformations of cardiac chambers and connections |  | Q20 |
|  | Common arterial trunk | 745.0 | Q20.0 |
|  | Double outlet right ventricle | 745.11 | Q20.1 |
|  | Double outlet left ventricle | 745.19 | Q20.2 |
|  | Discordant ventriculoarterial connection | 745.10; 745.19 | Q20.3 |
|  | Double inlet ventricle | 745.3 | Q20.4 |
|  | Discordant atrioventricular connection | 745.12 | Q20.5 |
|  | Isomerism of atrial appendages | 745.8 | Q20.6 |
|  | Other congenital malformations of cardiac chambers and connections | 745.19; 745.7; 745.8 | Q20.8 |
|  | Congenital malformation of cardiac chambers and connections, unspecified | 746.9 | Q20.9 |
|  | Congenital malformations of cardiac septa |  | Q21 |
|  | Ventricular septal defect | 745.4 | Q21.0 |
|  | Atrial septal defect | 745.5 | Q21.1 |
|  | Atrioventricular septal defect | 745.60; 745.61; 745.69 | Q21.2 |
|  | Tetralogy of Fallot | 745.2 | Q21.3 |
|  | Aortopulmonary septal defect | 745.8 | Q21.4 |
|  | Other congenital malformations of cardiac septa | 745.8 | Q21.8 |
|  | Congenital malformation of cardiac septum, unspecified | 745.9 | Q21.9 |
|  | Congenital malformations of pulmonary and tricuspid valves |  | Q22 |
|  | Pulmonary valve atresia | 746.01 | Q22.0 |
|  | Congenital pulmonary valve stenosis | 746.02 | Q22.1 |
|  | Congenital pulmonary valve insufficiency | 746.09 | Q22.2 |
|  | Other congenital malformations of pulmonary valve | 746.00 | Q22.3 |
|  | Congenital tricuspid stenosis | 746.1 | Q22.4 |
|  | Ebstein's anomaly | 746.2 | Q22.5 |
|  | Hypoplastic right heart syndrome | 746.1 | Q22.6 |
|  | Other congenital malformations of tricuspid valve | 746.1 | Q22.8 |
|  | Congenital malformation of tricuspid valve, unspecified | 746.1 | Q22.9 |
|  | Congenital malformations of aortic and mitral valves |  | Q23 |
|  | Congenital stenosis of aortic valve | 746.3 | Q23.0 |
|  | Congenital insufficiency of aortic valve | 746.4 | Q23.1 |
|  | Congenital mitral stenosis | 746.5 | Q23.2 |
|  | Congenital mitral insufficiency | 746.6 | Q23.3 |
|  | Hypoplastic left heart syndrome | 746.7 | Q23.4 |
|  | Other congenital malformations of aortic and mitral valves | 746.89 | Q23.8 |
|  | Congenital malformation of aortic and mitral valves, unspecified | 746.89 | Q23.9 |
|  | Other congenital malformations of heart |  | Q24 |
|  | Dextrocardia | 746.87 | Q24.0 |
|  | Levocardia | 746.87 | Q24.1 |
|  | Cor triatriatum | 746.82 | Q24.2 |
|  | Pulmonary infundibular stenosis | 746.83 | Q24.3 |
|  | Congenital subaortic stenosis | 746.81 | Q24.4 |
|  | Malformation of coronary vessels | 746.85 | Q24.5 |
|  | Congenital heart block | 746.86 | Q24.6 |
|  | Other specified congenital malformations of heart | 746.84; 746.87; 746.89 | Q24.8 |
|  | Congenital malformation of heart, unspecified | 746.9 | Q24.9 |
|  | Congenital malformations of great arteries |  | Q25 |
|  | Patent ductus arteriosus | 747.0 | Q25.0 |
|  | Coarctation of aorta | 747.10 | Q25.1 |
|  | Atresia of aorta | 747.11; 747.22 | Q25.2 |
|  | Interruption of aortic arch |  | Q25.21 |
|  | Other atresia of aorta |  | Q25.29 |
|  | Supravalvular aortic stenosis | 747.22 | Q25.3 |
|  | Other congenital malformations of aorta | 747.20; 747.21; 747.29 | Q25.4 |
|  | Congenital malformation of aorta unspecified |  | Q25.40 |
|  | Absence and aplasia of aorta |  | Q25.41 |
|  | Hypoplasia of aorta |  | Q25.42 |
|  | Congenital aneurysm of aorta |  | Q25.43 |
|  | Congenital dilation of aorta |  | Q25.44 |
|  | Double aortic arch |  | Q25.45 |
|  | Tortuous aortic arch |  | Q25.46 |
|  | Right aortic arch |  | Q25.47 |
|  | Anomalous origin of subclavian artery |  | Q25.48 |
|  | Other congenital malformations of aorta |  | Q25.49 |
|  | Atresia of pulmonary artery | 747.31 | Q25.5 |
|  | Stenosis of pulmonary artery | 747.39 | Q25.6 |
|  | Other congenital malformations of pulmonary artery |  | Q25.7 |
|  | Coarctation of pulmonary artery | 747.31 | Q25.71 |
|  | Congenital pulmonary arteriovenous malformation | 747.32 | Q25.72 |
|  | Other congenital malformations of pulmonary artery | 747.39 | Q25.79 |
|  | Other congenital malformations of other great arteries | 747.29 | Q25.8 |
|  | Congenital malformation of great arteries, unspecified | 747.29 | Q25.9 |
|  | Congenital malformations of great veins |  | Q26 |
|  | Congenital stenosis of vena cava | 747.49 | Q26.0 |
|  | Persistent left superior vena cava | 747.49 | Q26.1 |
|  | Total anomalous pulmonary venous connection | 747.41 | Q26.2 |
|  | Partial anomalous pulmonary venous connection | 747.42 | Q26.3 |
|  | Anomalous pulmonary venous connection, unspecified | 747.42 | Q26.4 |
|  | Anomalous portal venous connection | 747.61 | Q26.5 |
|  | Portal vein-hepatic artery fistula | 747.61 | Q26.6 |
|  | Other congenital malformations of great veins | 747.49 | Q26.8 |
|  | Congenital malformation of great vein, unspecified | 747.40 | Q26.9 |
|  | Other congenital malformations of peripheral vascular system | 747.5  747.62  747.62  747.89  747.63  747.64  747.61  747.62  747.69  747.89  747.69  747.60 | Q27.x |
|  | Other congenital malformations of circulatory system | 747.89  747.89  747.81  747.81  747.89  747.9 | Q28.x |
| Respiratory failure | Other abnormalities of breathing | 784.99  786.09  786.9 | R06.89 |
|  | Hypoxemia | 518.51  518.81 | R09.02 |
|  | Respiratory failure, unspecified, unspecified whether with hypoxia or hypercapnia | 518.81 | J96.90 |
|  | Respiratory failure, unspecified with hypercapnia | 518.81 | J96.92 |
| Neuromuscular disorders | Anencephaly and similar malformations | 740.x | Q00.x |
|  | Encephalocele | 742.0 | Q01.x |
|  | Microcephaly | 742.1 | Q02.x |
|  | Congenital hydrocephalus | 742.3 | Q03.x |
|  | Other congenital malformations of brain | 742.2; 742.4; 742.9 | Q04.x |
|  | Spina bifida | 741.x | Q05.x |
|  | Other congenital malformations of spinal cord | 742.51; 742.59; 742.53; 742.9 | Q06.x |
|  | Other congenital malformations of nervous system | 741.90; 741.00; 742.8; 742.9 | Q07.x |
|  | Myalgia and myositis, unspecified | 729.1 | M60.9; M79.1; M79.7 |
|  | Diseases of the nervous system | 320.xx- 359.xx | G00.xx-G99.xx |
| Bronchopulmonary dysplasia | Bronchopulmonary dysplasia originating in the perinatal period | 770.7 | P27.1 |
| Down syndrome | Down syndrome |  | Q90 |
|  | Trisomy 21, nonmosaicism (meiotic nondisjunction) | 758.0 | Q90.0 |
|  | Trisomy 21, mosaicism (mitotic nondisjunction) | 758.0 | Q90.1 |
|  | Trisomy 21, translocation | 758.0 | Q90.2 |
|  | Down syndrome, unspecified | 758.0 | Q90.9 |
| Immunodeficiency | Immunodeficiency with predominantly antibody defects | 279.04  279.00  279.01  279.03  279.02  279.05  279.19  279.09  279.19  279.19 | D80.x |
|  | Combined immunodeficiencies | 279.2  279.2  279.2  277.2  279.13  277.2  279.2  279.2  277.6  266.2  266.2  279.2  279.2 | D81.x |
|  | Immunodeficiency associated with other major defects | 279.12  279.11  279.8  279.8  279.8  279.8  279.8 | D82.x |
|  | Common variable immunodeficiency | 279.06  279.10  279.06  279.06  279.06 | D83.x |
|  | Other immunodeficiencies | 279.8  277.6  279.8  279.3  279.3 | D84.x |
|  | Other disorders involving the immune mechanism, not elsewhere classified | 273.0  273.2  273.1  289.89  279.8  279.51  279.52  279.53  279.50  279.41  279.8  279.9 | D89.x |
|  | Human immunodeficiency virus (HIV) disease | 042 | B20 |
| Velo-cardio-facial syndrome | Velo-cardio-facial syndrome | 758.32 | Q93.81 |
| Congenital anomalies of respiratory system | Congenital anomalies of respiratory system | 748.x | Q30.x – Q34.x |
| Other congenital musculoskeletal anomalies | Other congenital musculoskeletal anomalies | 756.x | Q75.x-Q79.x |
| Cystic fibrosis with pulmonary manifestations | Cystic fibrosis | 277.0 | E84 |
|  | Cystic fibrosis with pulmonary manifestations | 277.02 | E84.0 |
|  | Cystic fibrosis with intestinal manifestations |  | E84.1 |
|  | Meconium ileus in cystic fibrosis | 277.01 | E84.11 |
|  | Cystic fibrosis with other intestinal manifestations | 277.03 | E84.19 |
|  | Cystic fibrosis with other manifestations | 277.09 | E84.8 |
|  | Cystic fibrosis, unspecified | 277.00 | E84.9 |

Listing S2 List of ICD-9-MC and ICD-10-ES codes used as “severity markers”

| **Definition** | **ICD-9-MC** | **ICD-10-ES** |
| --- | --- | --- |
| Oxygenation | 93.96 | 3E0F7GC |
| Mechanical ventilation – non-invasive | 93.90 | 5A0.xxxx |
| Mechanical ventilation – invasive | 96.7x | 5A1.xxxx |
| Other abnormalities of breathing | 784.99  786.09  786.9 | R06.89 |
| Hypoxemia | 518.51  518.81 | R09.02 |
| Respiratory failure, unspecified, unspecified whether with hypoxia or hypercapnia | 518.81 | J96.90 |
| Respiratory failure, unspecified with hypercapnia | 518.81 | J96.92 |

Listing S3 Evolution in the incidence rate of hospitalizations by respiratory syncytial virus definition and age group

|  | **RSV-specific** | | | | **RSV-specific & Bronchiolitis** | | | | | **RSV-specific & ALRI** | | | |
| --- | --- | --- | --- | --- | --- | --- | --- | --- | --- | --- | --- | --- | --- |
| Age group | **2015/16** | **2016/17** | **2017/18** | **Mean** | **2015/16** | **2016/17** | **2017/18** | **Mean** | **2015/16** | | **2016/17** | **2017/18** | **Mean** |
| [0-12[ months | 32.9 | 28.9 | 26.5 | **29.4** | 51.0 | 45.2 | 41.8 | **46.0** | 62.2 | | 54.7 | 49.7 | **55.5** |
| [12-24[ months | 4.2 | 3.8 | 3.3 | **3.8** | 5.9 | 5.0 | 4.5 | **5.1** | 19.1 | | 15.4 | 13.5 | **16.0** |
| [24-36[ months | 1.3 | 1.3 | 1.2 | **1.3** | 1.5 | 1.4 | 1.3 | **1.4** | 11.1 | | 8.7 | 7.4 | **9.0** |
| [36-60[ months | 0.2 | 0.3 | 0.3 | **0.3** | 0.3 | 0.3 | 0.3 | **0.3** | 4.7 | | 3.4 | 3.1 | **3.7** |
| [0-24[ months | 18.4 | 16.2 | 14.6 | **16.4** | 28.2 | 24.8 | 22.7 | **25.2** | 40.4 | | 34.8 | 31.3 | **35.5** |
| [24-60[ months | 0.6 | 0.6 | 0.6 | **0.6** | 0.6 | 0.6 | 0.6 | **0.6** | 6.7 | | 5.1 | 4.5 | **5.4** |
